# Guillain-Barré Syndrome With a Peculiar Course: A Case Report

**DOI:** 10.7759/cureus.14905

**Published:** 2021-05-08

**Authors:** Lucia Sur, Gabriel Samasca, Genel Sur, Remus Gaga, Cornel Aldea

**Affiliations:** 1 Pediatrics, Iuliu Hațieganu University of Medicine and Pharmacy, Cluj-Napoca, ROU; 2 Immunology, Iuliu Hațieganu University of Medicine and Pharmacy, Cluj-Napoca, ROU; 3 Pediatrics, Emergency Clinical Hospital for Children, Cluj-Napoca, ROU

**Keywords:** hypotonia, guillan-barre syndrome, nerves, peculiar course, intravenous immunoglobulins

## Abstract

Guillain-Barré syndrome (GBS) or acute inflammatory demyelinating polyradiculoneuropathy (AIDP) is a rare autoimmune disorder in which the body's immune system mistakenly attacks the nerves. In this report, we present a case of a 15-month-old girl who presented with an inability to walk and support the vertical and sitting positions, pain in the lower limbs accompanied by grimaces, muscular weakness, and agitation due to gait disturbances. This is a unique case in that GBS affected a previously healthy girl and was associated with pneumonia and anemia as the disease progressed, causing an intriguing diagnosis. Also, another remarkable aspect of our case is that complete recovery was achieved following intravenous immunoglobulin (IVIG) and anti-inflammatory treatment; our patient was able to walk again after receiving the first dose of IVIG.

## Introduction

Guillain-Barré syndrome (GBS), also known as acute inflammatory demyelinating polyradiculoneuropathy (AIDP), refers to a heterogeneous condition characterized by an acute non-febrile, post-infectious illness manifesting as ascending weakness, areflexia, and other sensory and motor abnormalities. Regarding the pathophysiology of the disease, the latest research in the field considers GBS as an autoimmune disease, often triggered by viral or bacterial infections caused by organisms such as *Campylobacter jejuni*, cytomegalovirus, Epstein-Barr virus, or *Mycoplasma pneumoniae*. Vaccinations against the flu, rabies, and meningitis have also been documented as possible causes. The diagnosis is often based on a progressive ascending weakness with areflexia. Also, findings from lumbar puncture, electrodiagnostic studies, or MRI (enhancement of the nerve roots with gadolinium) can support the diagnosis. The presence of serum ganglioside antibodies is another criterion for the diagnosis and evaluation of GBS. However, the value of these antibodies as a prognostic marker in children is still under evaluation.

The outcome of GBS is generally favorable in children, despite the long recovery period. The most effective form of therapy is intravenous immunoglobulin (IVIG) administration [1]. Despite the fact that GBS is often considered an easy diagnosis based on anamnesis and clinical examination, as well as laboratory and neurophysiological investigations [2], sometimes the heterogeneity of the disease can be intriguing. We report a case of GBS in a 15-month-old girl, which presented some unique challenges regarding the differential diagnosis.

## Case presentation

A 15-month-old girl, with normal psychomotor development for her age, was brought to Pediatric Clinic II (Cluj-Napoca, Romania) with complaints of inability to walk or support the vertical and sitting positions, pain with the appearance of grimaces, muscular weakness, and agitation. The current disease had been sudden in its onset and had started approximately a month before the presentation. The disease onset had been characterized by muscle aches and difficulty in walking. The patient’s mother had given her ibuprofen oral suspension 10 mg/kg per dose at home, which had partially relieved the symptoms. However, her condition had worsened after almost two weeks due to a relapse. On consulting the family doctor, an orthopedic examination had been recommended. However, the orthopedist had not established a final diagnosis. As the symptoms persisted, the girl was admitted to Pediatric Clinic II for further investigations, diagnosis, and treatment. The patient's father had hepatitis B. The pathological personal history was positive for an episode of acute laryngitis and atopic dermatitis.

At the time of admission to the hospital, the patient's general health status was found to be good; she was afebrile, with an uncharacteristic face, macular rash, and normal-colored mucous membrane and pharynx, and showed no signs of lymphadenopathy. The lung examination showed normal vesicular sounds, no crackles, rubs, wheezing, or rhonchi. The cardiovascular examination revealed regular rate and rhythm, with normal S1 and S2, without murmurs, rubs, or gallops, and the peripheral pulses were palpable bilaterally. The examination of the gastrointestinal system showed a soft, non-tender, and non-distended abdomen with no masses, no hepatosplenomegaly, no abdominal pain, no change in bowel habits, and normal bowel sounds. The genitourinary examination revealed no abnormalities.

The neurological examination findings were as follows: inability to walk or move the lower limbs; pain expressed by grimaces due to the palpation of the lower limbs; muscular hypotonia of the limbs, predominantly in the lower limbs; diminished deep tendon reflexes of the upper limbs and abolished deep tendon reflexes of the lower limbs; the bilateral extension of plantar cutaneous reflex; cranial nerves grossly intact; no decrease in sensation; no focal neurologic deficits; and no signs of meningeal irritation. Due to the suspicion for GBS, the patient was administered 1 g/kg IVIG in two doses, along with hydrocortisone hemisuccinate 50 mg/day. After the first dose of gamma globulins, the patient resumed her gait: about two hours after receiving the treatment, she was able to get down from the bed and realize the active movements she previously had, although still experiencing a slight pain in mobilization. After the second dose, neurological dynamics normalized completely.

After administering the first dose of gamma globulins, the neurological examination was repeated, and it revealed that the patient was able to walk on the peaks with support; it also showed muscular hypotonia in the limbs (predominantly of the lower limbs), bilateral dorsal flexion angle of the foot of 40-60 degrees, 2+, symmetric, deep tendon reflexes, and the bilateral extension of cutaneous plantar reflex. Based on the neurological examination, the pediatric neurologist provided some possible diagnoses, along with some recommendations: episodic, periodic paralysis; evaluation for arthritis; periodic paralysis with hyper- or hypokalemia; benign paroxysmal vertigo with indications for cerebral MRI examination; and alternating hemiplegia (mitochondrial disease) that warrants a funduscopic examination and a cardiac consult. As per the mother's assertions, the patient had presented with dorso-lumbar hypotonia and tenderness of the lumbar spine; hence, imaging-based investigations were recommended. Corticosteroid therapy, as well as Carnil 1 g 1/2 vials/day, was also recommended.

During the hospital admission, the laboratory investigations revealed hypochromic microcytic anemia, mild monocytosis, upper-limit blood levels of alkaline phosphatase, high blood levels of creatine phosphokinase, electrolytes within the normal range, normoglycemia, lactate dehydrogenase (LDH) within normal limits, negative HBs antigens, no immunoglobulin M (IgM) and IgG anti-Epstein Barr virus antibodies, negative IgM anti-cytomegalovirus antibodies, positive IgG anti-cytomegalovirus antibodies, and no anti-*Borrelia burgdorferi* antibodies. Blood samples were also collected for the genetic tests for the mutation of survival genes motoneuron (SMN) 1 and SMN 2.

After one week of treatment, laboratory investigations were repeated and showed an inflammatory syndrome [erythrocyte sedimentation rate (ESR): 46 mm/h (normal range: 0-14 mm/h)], hypochromic microcytic anemia, C3 complement within the normal range, normal levels of immunogram, with G hyperimmunoglobulinemia, and negative IgM anti-toxoplasma antibodies and positive IgG anti-toxoplasma antibodies. The peripheral blood film showed lymphocytosis with variable cytoplasmic basophilia and vacuolated monocytes. The electron-microscopic study revealed mild anisocytosis with normo-, micro-, and rare macrocytes; variable hypochromia; mild polychromatophilia; and rare codocytes and ovalocytes. Four days later, repeated laboratory tests revealed the persistence of inflammatory syndrome (ESR: 57 mm/h), hypochromic microcytic anemia, and lymphopenia, without any other changes in the paraclinical parameters.

The electro-neurophysiological tests were positive for GBS, which explained the slowness of the nerve conduction in the limbs.

**Figure 1:**
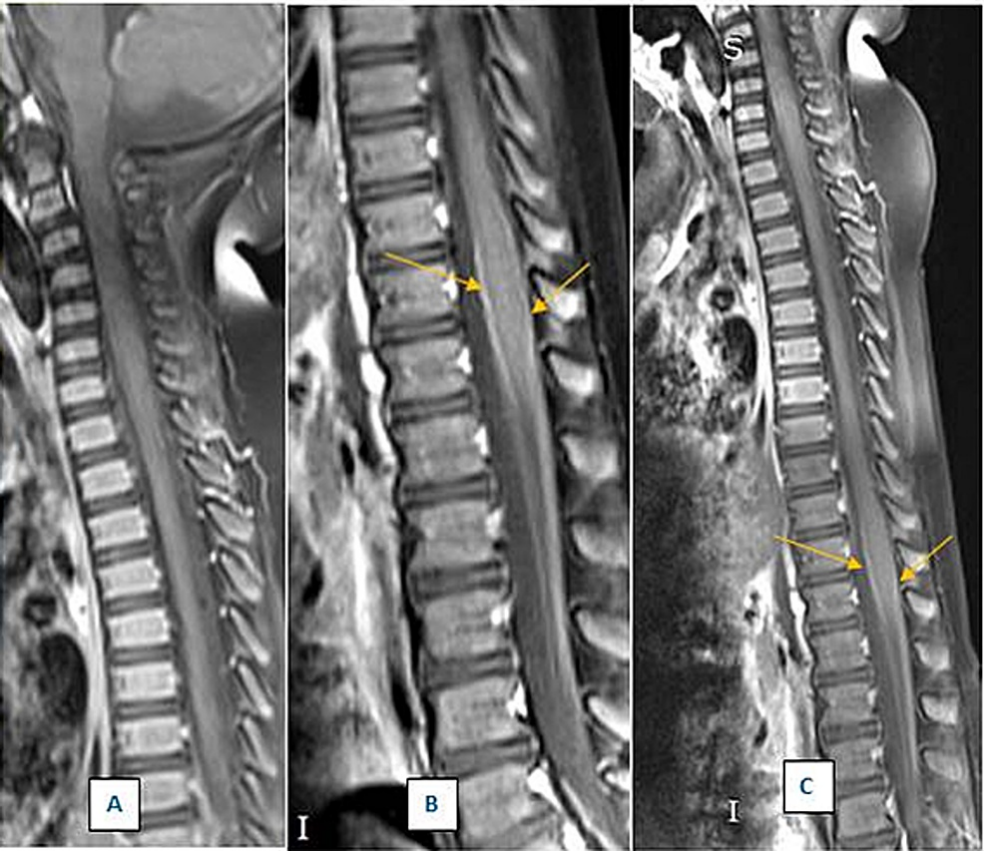
Dorso-lumbar MRI of the spine A. Dorsal spine in the sagittal plane; T1 sequence with contrast substance and fat impregnation, with normal aspect B. Lumbar spine in the sagittal plane; T1 sequence with contrast substance and fat impregnation, with peripheral capture in the terminal medullaris conus (the white line on the limits of the medullaris conus is indicated by yellow arrows) C. Restored image of the dorsal and lumbar parts of the spine (a composite of images 1A and 1B) MRI: magnetic resonance imaging

None of the following were observed on the cerebral and dorsal-lumbar MRI: heterosignal lesions in the supra- or infratentorial cerebral substance, acute or cerebral ischemia or intracranial hemorrhagic collections, deviation of the midline structures, distension or asymmetry of the ventricular system, and tumescence of the bilateral mucosa of the maxillary and ethmoidal sinuses. Normal pneumatization of the temporo-mastoid cells was noted. The following aspects were also observed: minor scoliotic dorsal-lumbar dextroconcave position, no pathological changes in the spinal structure and cervical-dorsal-lumbar medullary cord, and the medullaris conus near the lumbar vertebral level L1. Contrast MRI revealed peripheral capture in the terminal medullaris conus and in the several spinous process roots, which was compatible with the suspicion for GBS (Figure 1). There were no other pathological contrast captures at the other medullary levels, and no obvious pathological changes in the intervertebral discs were observed. On the dorsal column sequences, the lung was partially visualized and pulmonary condensation (more likely pneumonia) was noted at the lumbar-sacral-dorsal level. In conclusion, the MRI indicated the aspect of the contrast capture at the conus terminalis level, as well as right pulmonary condensation (pneumonia), as had been suspected.

The echocardiographic examination revealed minor hypertrophied left ventricle, with good global kinetics, minor mitral insufficiency, with no hemodynamic significance, normal systolic and diastolic function, no pericardial collection, and no sign of pulmonary hypertension. An electrocardiogram showed sinus rhythm, heart rate of 118 beats per minute, ax QRS of 49 degrees, corrected QT of 377 ms, and no pathological modifications. A re-examination was recommended if required. Based on the clinical and paraclinical investigations, the diagnosis of GBS was confirmed.

Treatment with 25-mg hydrocortisone and Ig (1 g/kg/day-2 g/total dose administered) was initiated at the hospital. The patient's status improved with the treatment, leading to the progressive recovery of muscle tonus, with the possibility of the improvement of the gait, which was initially done with support and subsequently on the peaks, with minor imbalance. The administration of corticosteroids and Ig was suspended. After almost a week, the patient again presented with hypotonia of the limbs, inability to walk, and psychomotor agitation, which was treated with 25 mg of hydrocortisone. Laboratory investigations are relevant to monitor the persistence of inflammatory syndrome, anemia, and changes in the peripheral blood smear. Due to the neurological involvement, we added Carnil 100 mg/ml to the patient’s treatment regimen. We gradually decreased the dose of hydrocortisone to 3 x 25 mg/day, and subsequently to 2 x 25 mg/day until the drug was stopped after 10 days of treatment.

After 10 days of hydrocortisone treatment, the patient had a dry cough and an influenced general health status, and laboratory analyses revealed inflammatory syndrome, neutrophilia, minor monocytosis, and normochromic normocytic anemia. We decided to introduce the antibiotic ceftriaxone (Cefort) 1 g, the antipyretic metamizolum natrium (Algocalmin) 1 g/2 ml, and probiotic (Levurin) treatment in the patients’ treatment plan. She was discharged with the following recommendations: Carnil 100 mg/ml 1/2 vial/day for one month, ibuprofen (Nurofen) 100 mg/ml syrup 2 x 5 ml/day for two weeks, and vitamin B complex syrup 5 ml/day for 25 days; her prognosis was favorable. At a follow-up one month later, an improved general health status with remission of the symptomatology and progressive recovery of the gait was observed.

## Discussion

In this case, we ruled out any suspicion for neuromuscular diseases, such as spinal amyotrophy (SMA), which is a progressive neurodegenerative disease [3]. In GBS, the weakness ascends acutely from distal sites, which differentiates it from the more insidious onset and proximal location associated with SMA.

Another pathology that we considered and excluded was hyperkalemia [4]. Hyperkalemia manifests clinically with acute neuromuscular paralysis, which can simulate GBS. Some of the mechanisms behind this abnormality are as follows: genetic defects in the sodium channel, renal dysfunction, potassium retaining drugs, Addison's disease, etc. Clinical characteristics of GBS have been addressed in a number of publications. However, electrophysiological evaluations of these patients during neuromuscular paralysis have shown a loss of myelin, which is associated with the slowing of conduction velocity. The clinical features and electrophysiological abnormalities are more difficult to diagnose in secondary hyperkalemia because the underlying cause is often unknown.

We also ruled out the diagnosis of congenital muscular dystrophy [5]. Duchenne and Becker muscular dystrophies should be considered as they present with progressive weakness after normal development in patients in the age range (two to three years) that is close to our patients’ age. Mitochondrial myopathies may also occur at any age in childhood, but the symptomatology is different, including ptosis or sensory loss that are not accompanied by progressive ascending paralysis. Also, congenital neuropathies such as Charcot-Marie-Tooth disease are characterized by an important sensory component evaluated on electrodiagnostic testing. Cognitive dysfunction is also a comorbidity associated with muscular dystrophies, which would not be expected to be present in GBS [6].

Another diagnosis that we considered was infections, such as Lyme disease (infection with *Borrelia burgdorferi*), toxoplasmosis, cytomegalovirus infection, or infection with Epstein Barr virus [7]. With the advent of poliovirus vaccination and the eradication of poliomyelitis, GBS is currently the most common cause of acute motor paralysis in children. Despite the decline in poliovirus infections, other enteroviruses and West Nile virus can cause often asymmetric paralysis in the lower extremities.

We also excluded the following diagnoses: acute cerebellar ataxia, benign paroxysmal vertigo or vestibular migraine, and drug poisoning [8].

## Conclusions

The peculiarity of our case is that GBS affected a previously healthy girl and it was associated with pneumonia and anemia as the disease progressed; the patient achieved complete recovery following IVIG and anti-inflammatory treatment. Another unique aspect of this case is that our patient regained the ability to walk after receiving the first dose of IVIG.
